# Chemokines during anaphylaxis: the importance of CCL2 and CCL2-dependent chemotactic activity for basophils

**DOI:** 10.1186/s13601-020-00367-2

**Published:** 2020-12-15

**Authors:** Romana Vantur, Marusa Rihar, Ana Koren, Matija Rijavec, Peter Kopac, Urska Bidovec-Stojkovic, Renato Erzen, Peter Korosec

**Affiliations:** 1 University Clinic of Respiratory and Allergic Diseases Golnik, Golnik 36, 4204 Golnik, Slovenia; 2 Biotechnical Faculty, University of Ljubljana, Ljubljana, Slovenia; 3 Medical Faculty, University of Ljubljana, Ljubljana, Slovenia

**Keywords:** Anaphylaxis, Chemokines, CCL2, Tryptase, Basophils, Chemotaxis, Migration

## Abstract

**Background:**

The role of chemokines in anaphylaxis is unclear.

**Methods:**

We prospectively recruited 49 patients presenting to the emergency department with an acute episode of anaphylaxis and 28 healthy subjects. We measured serum levels of the chemokines CCL2, CCL5, CCL7, CCL8, CCL11, CCL13, CCL17, CCL21, CCL22, CCL24, and CCL26, tryptase, the absolute number of circulating basophils, monocytes, lymphocytes, and PMNs, and whole blood *FCER1A*, *CPA3* and *HDC* gene expression at two time points: during the anaphylactic episode and in convalescent samples collected approximately 3 months later. We then investigated the in vitro chemotactic activity of chemokines induced during anaphylaxis for the in vitro migration of the corresponding cells.

**Results:**

Only CCL2 chemokine levels were significantly increased in anaphylaxis samples (median 514 pg/ml) compared to convalescent samples (284 pg/ml, *P* < 0.0001) and healthy subjects (279 pg/ml, *P* < 0.0001); there was no significant difference in any of the other chemokines. There was a significant positive correlation between the rates of increase of serum CCL2 (median [range]: 106.0% [− 44.7% to 557.4%]) and tryptase (133.8% [− 6.6% to 893.4%]; r = 0.68, P < 0.0001) and between the acute concentration of serum CCL2 and the acute concentration of serum tryptase (r = 0.77, P < 0.0001). The number of circulating basophils, but not other blood cells, significantly decreased during anaphylaxis (median 5.0 vs. 19.1 cells/µl in convalescent samples; *P* < 0.0001); a decrease in whole-blood gene expression of basophil markers (*P* ≤ 0.0018) confirmed these changes. Anaphylactic serum enhances the in vitro migration of basophils via CCL2-dependent chemotactic activity; in contrast, no CCL2-dependent chemotactic activity was observed for convalescent samples.

**Conclusions:**

Our findings imply an important and specific role for CCL2-mediated chemotactic activity in the pathophysiology of human anaphylaxis.

## Background

Anaphylaxis is a potentially life-threatening, rapidly progressing systemic hypersensitivity reaction, often following exposure to a small amount of allergen, including insect venom, foods, and medications [1]. The major immunopathogenesis and pathophysiological mechanisms of anaphylaxis involve IgE antibodies, effector mast cells, basophils and the mediators histamine, platelet-activating factor, and cysteinyl leukotrienes [2–4]. However, anaphylaxis also induces changes in other mediators, including tryptase [5–7], prostaglandins [8], cytokines [6, 9], and chemokines [7]. A recent study demonstrated an increase in CCL2 (C-C Motif Chemokine Ligand 2) levels during human anaphylaxis [7], with no changes demonstrated for the chemokines CCL5 [7] and CCL11 [6, 7]. The chemokines CCL2, CCL5, and CCL11 are involved in basophil migration, with the CCR2 ligand CCL2 and the CCR3 ligand CCL11 eliciting the most potent migratory responses [10]. Chemokines that diffuse out from the site of release and form a concentration gradient to which leukocytes respond and migrate might be involved in the recruitment of basophils [4] or other potential effector cells of anaphylaxis, including monocytes/macrophages [11], neutrophils [12, 13], and platelets [14], to the inflamed tissue sites.

In addition to CCL2 [7], several other chemokines might be involved in different allergic diseases. For monocyte chemoattractant proteins (MCPs), an increase in CCL7 levels in allergic conjunctivitis [15], an increase in CCL8 levels in chronic/eosinophilic atopic dermatitis [16], and an increase in CCL13 levels in allergic rhinitis [17] were demonstrated. For eotaxin chemokines, CCL11 and CCL24 levels were increased in asthma and allergic rhinitis [18], and CCL26 levels were increased in allergic rhinitis [17] and atopic skin inflammation [19]. CCL5, CCL17, CCL21, and CCL22 were also reported to be involved in asthma, atopic dermatitis, allergic rhinitis and drug hypersensitivity syndrome [20–25].

Given that different chemokines might be important in allergic diseases, we performed a series of interlinked studies to better understand the role of chemokines and their chemotactic activity in anaphylaxis. In a prospective study during and after anaphylaxis caused by *Hymenoptera* venom, medication, food, or other (idiopathic), we investigated the serum concentration of a large panel of different chemokines (CCL2, CCL5, CCL7, CCL8, CCL11, CCL13, CCL17, CCL21, CCL22, CCL24, and CCL26), which were previously described in allergic diseases [6, 7, 15–25]. In whole blood samples, we measured the absolute numbers of circulating basophils, monocytes, lymphocytes, and polymorphonuclear leukocytes (PMNs) and the gene expression of the basophil markers *FCER1A*, carboxypeptidase A3 (*CPA3*) and L-histidine decarboxylase (*HDC*). We then proceeded to analyse the chemotactic activity of chemokines that are induced during anaphylaxis in the controlled setting of a cellular in vitro migration assay.

## Methods

### Study participants

We prospectively recruited 49 patients (24 females; age, 19–82 years; Table 1) presenting to the Emergency Department of University Hospital Golnik, Slovenia, with an acute episode of anaphylaxis (January 2017 to October 2019). The reaction was caused by an insect sting in 26 patients, by medication in 14 patients, by food in 5 patients and by other triggers in 4 patients (idiopathic in 2 patients and infection in 2 patients); 21 experienced Mueller [26] grade IV, 16 experienced grade III, 6 experienced grade II, and 6 experienced grade I reactions (Table 1). We collected blood samples during the reaction (at presentation to the emergency department; in all patients we routinely measured acute serum tryptase, Table 1) and convalescent samples after the anaphylactic episode. None of those patients were included in our previous study of anaphylaxis [7]. The median time from the onset of symptoms to sample collection was 100 min (range 30 to 240 min); convalescent samples were collected a median of 80 days after the anaphylactic episode (Table 1). In 30 patients, we measured serum chemokines and tryptase, and in 26 patients, we measured blood absolute cell counts and gene expression (of those 41 patients, 15 were in both subgroups); for comparison, we recruited 20 healthy subjects (13 females; age, 20–76 years) (Additional file 1: Table S1). The serum of 8 anaphylactic patients and isolated basophils of 8 healthy subjects (4 females; age, 25–44 years) were used for in vitro migration experiments (Additional file 1: Table S1).

**Table 1:** Characteristics of the patients

	Patients with an acute episode of anaphylaxis (n = 49)
Sex, n (%)	24 female (49.0) 25 male (51.0)
Age years, average (range)	50 (19–82)
Trigger, n (%)	
*Hymenoptera* venom	26 (53.1)
Medication	14 (28.6)
Food	5 (10.2)
Other	4 (8.2)
Time from the onset of anaphylactic symptoms to sample collection at presentation to the emergency department, median (IQR) in minutes	100 (69)
Convalescent sampling after anaphylactic episode, median (IQR) in days	80 (93)
Emergency treatment, n (%)	
Epinephrine	25 (51.0)
Corticosteroids	43 (87.8)
Grade of reaction, n (%)	
Mueller I	6 (12.2)
Mueller II	6 (14.6)
Mueller III	16 (32.7)
Mueller IV	21 (42.9)
Symptoms, n (%)	
Any skin feature	44 (89.8)
Any gastrointestinal feature	15 (30.6)
Any respiratory feature	26 (53.1)
Hypoxemia (SpO2 ≤ 92%)	7 (14.3)
Any cardiovascular feature	24 (49.0)
Hypotension (SBP < 90 mmHg)	19 (38.8)
Acute tryptase > 11.4 µg/l, n (%)	32 (65.3)

SBP systolic blood pressure

Ethical approval was obtained from the Slovenian National Medical Ethics Committee. All subjects provided written informed consent.

### Serum chemokines and tryptase, absolute cell counts, and gene expression

We measured serum concentrations of the MCP chemokines CCL2/MCP-1, CCL7/MCP-3, CCL8/MCP-2, and CCL13/MCP-4; the eotaxin chemokines CCL11/Eotaxin, CCL24/Eotaxin-2 and CCL26/Eotaxin-3; and the chemokines CCL5/Rantes, CCL17/TARC, CCL21/SLC and CCL22/MDC using ELISAs according to the manufacturers’ instructions (Quantikine R&D Systems, Minneapolis, MN, USA; Abnova, Taipei, Taiwan; and Biolegend, San Diego, California, CA, USA). We measured serum tryptase (ImmunoCAP, Thermo Fisher, Uppsala, Sweden), and concentrations that exceeded 11.4 µg/l were considered increased. Enumeration of circulating basophils (CD123+HLA-DR− cells), monocytes, lymphocytes, and PMNs in fresh whole blood was performed via flow cytometry as previously described [7, 27]. *FCER1A*, *CPA3,* and *HDC* gene expression was analysed in whole blood samples (PAXgene, PreAnalytiX, Hombrechtikon, Switzerland) as previously described [7, 27]. Detailed methods are provided in Additional file 1.

### Cell separation and in vitro migration assays

CCR2 surface expression on basophils of healthy donors was determined by anti-CCR2 mAb (CD192; Miltenyi Biotec, Bergisch Gladbach, Germany) staining, and basophils were isolated from peripheral whole blood by negative immunomagnetic selection (Miltenyi Biotec, Bergisch Gladbach, Germany) and exactly quantified by absolute flow cytometry basophil counts as previously described (CD123+HLA-DR− cells and microbeads) [7, 27]. For the migration assay, we used modified Boyden chamber and polycarbonate membrane cell culture inserts (Corning Inc., New York, USA). A total of 20,000 basophils were added to the upper wells, and the samples to be tested were placed in the lower wells. After incubation for 30 to 150 min at 37 °C, we collected the cells that had migrated to the lower wells and quantified them by absolute basophil counts [7, 27]. Basophil migration was calculated by using the following equation:

$$ {\text{Basophil}}\;{\text{migration}}\left( \% \right) = \left( {{\text{absolute}}\;{\text{number}}\;{\text{of}}\;{\text{migrated}}\;{\text{basophils}}/{\text{absolute}}\;{\text{number}}\;{\text{of}}\;{\text{seeded}}\;{\text{basophils}}} \right) \times 100. $$

For control experiments, we used recombinant CCL2 (Thermo Fisher Scientific, Massachusetts, USA). To block CCL2, we used anti-CCL2 neutralizing antibodies (Sigma-Aldrich, Missouri, USA). All experiments were independently performed in triplicate. Detailed methods are described in Additional file 1.

### Statistical analysis

The distribution of the data was assessed using the D’Agostino test. As the majority of the data were nonparametric, we used the Wilcoxon or Mann–Whitney test. P values were Bonferroni-corrected for the complete set of 19 variables, and a *P*-value < 0.0026 was accepted as significant. For the cellular in vitro migration assay, all experiments were independently performed in triplicate, and the results were compared with a t-test; a *P*-value < 0.05 was accepted as significant. To quantify associations between variables, we used Spearman or Pearson correlation. All analyses were performed using GraphPad Prism (GraphPad Software, La Jolla, CA, USA).

## Results

### The chemokine CCL2, but not other chemokines, is significantly increased during anaphylactic episodes

#### MCP chemokines CCL2/MCP-1, CCL7/MCP-3, CCL8/MCP-2, and CCL13/MCP-4

**Fig. 1:**
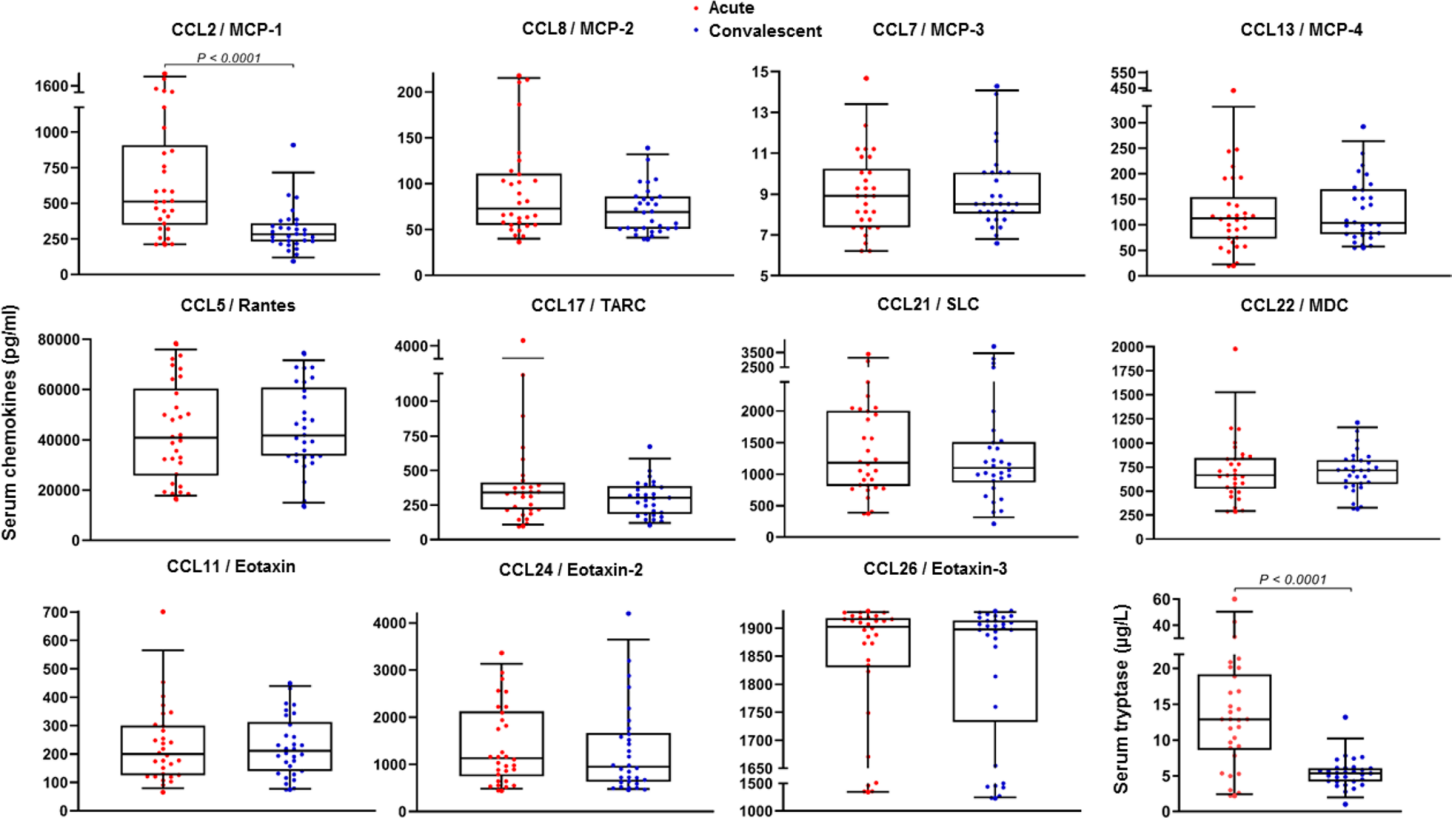
Serum CCL2, CCL5, CCL7, CCL8, CCL11, CCL13, CCL17, CCL21, CCL22, CCL24, CCL26, and tryptase levels in 30 patients during an acute anaphylactic episode and in convalescent samples collected later

**Fig. 2:**
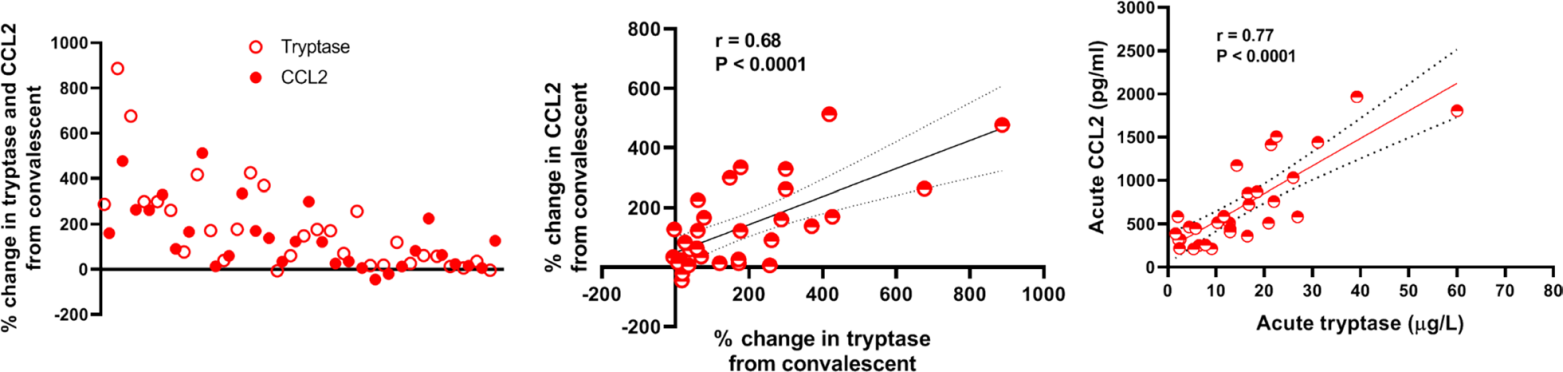
Comparison and correlation between serum CCL2 and tryptase levels in 30 patients with acute anaphylactic episodes

CCL2 concentrations measured during the anaphylactic episode (median 514 pg/ml) were significantly higher than those measured in convalescent serum samples collected later (median 80 days; 284 pg/ml; *P* < 0.0001; Fig. 1 and Table 2). This marked increase (median 106.0%; range: − 44.7% to 557.4%) was evident in 28/30 patients (Fig. 2). Serum CCL2 levels during the acute episode were also significantly higher than those observed in healthy subjects (median 279 pg/ml, *P* < 0.0001); however, CCL2 levels in convalescent samples showed no difference in comparison to CCL2 levels in healthy subjects (Fig. 1 and Table 2). Other MCP chemokines, including CCL7 (median 8.9, 8.5 and 8.1 pg/ml), CCL8 (73, 69 and 70 pg/ml) and CCL13 (113, 104 and 123 pg/ml), showed no differences during anaphylaxis or later compared to healthy subjects (Fig. 1 and Table 2).

**Table 2:** Laboratory data of anaphylactic patients and healthy subjects

Variables median (IQR)	Patients with an acute episode of anaphylaxis (n = 49)^a^		Healthy subjects (n = 28)^b^	P-value		
	Acute sampling	Convalescent sampling		Acute vs. convalescent	Acute vs. healthy subjects	Convalescent vs. healthy subjects
Serum chemokines (pg/ml)						
CCL2/MCP-1	514 (561)	284 (128)	279 (147)	**< 0.0001**	**< 0.0001**	0.9726
CCL7/MCP-3	8.9 (2.9)	8.5 (2.1)	8.1 (1.4)	0.7549	0.1636	0.1038
CCL8/MCP-2	73 (56)	69 (35)	70 (19)	0.1579	0.1425	0.4226
CCL13/MCP-4	113 (81)	104 (88)	123 (103)	0.9032	0.3367	0.7648
CCL11/Eotaxin	200 (174)	211 (174)	160 (60)	0.2710	0.1641	0.0555
CCL24/Eotaxin-2	1124 (1377)	946 (1036)	1159 (1561)	0.0940	0.8665	0.5919
CCL26/Eotaxin-3	1903 (88)	1898 (181)	1877 (155)	0.0412	0.1746	0.4640
CCL5/Rantes	40,907 (34,588)	41,795 (27,011)	46,993 (33,574)	0.6669	0.7485	0.9659
CCL17/TARC	342 (194)	303 (203)	344 (180)	0.0148	0.7093	0.1931
CCL21/SLC	1179 (1193)	1099 (645)	938 (417)	0.2206	0.3376	0.4175
CCL22/MDC	666 (321)	717 (251)	766 (260)	0.7045	0.8014	0.5408
Serum tryptase						
µg/l	12.9 (10.6)	5.3 (2.0)	3.3 (2.0)	**< 0.0001**	**< 0.0001**	**0.0005**
> 11.4 µg/l, n (%)	18 (60)	1 (3.3)	0 (0)	**< 0.0001**	**< 0.0001**	1
Absolute cell count (cells/µl)						
Basophils	5.0 (7.3)	19.1 (10.9)	–	**< 0.0001**	–	–
Monocytes	396 (163)	441 (108)	–	0.8417	–	–
Lymphocytes	1378 (1219)	1461 (470)	–	0.3904	–	–
PMNs	3881 (2873)	2875 (1144)	–	0.0388	–	–
Gene expression in whole blood (fold change)						
*FCER1A*	0.11 (0.24)	0.40 (0.64)	–	**0.0002**	–	–
*CPA3*	0.22 (0.51)	0.72 (1.16)	–	**0.0002**	–	–
*HDC*	0.11 (0.30)	0.38 (0.57)	–	**0.0018**	–	–
Serum of patients used for in vitro migration assays						
Tryptase (µg/l)	22.6 (16.5)	6.4 (6.2)	4.3 (5.8)	**0.0002**	**0.0003**	0.5358
> 11.4 µg/l, n (%)	8 (100)	0 (0)	0 (0)	**0.0002**	**0.0002**	1
CCL2 (pg/ml)	1964 (1114)	278 (175)	302 (76)	**0.0002**	**0.0003**	0.3969
Basophils of healthy donors used for the in vitro migration assays						
Absolute count (cells/µl)	–	–	18.3 (6.3)	–	–	–
CCR2 expression MFI	–	–	353 (422)	–	–	–
% CCR2 + basophils	–	–	94 (27)	–	–	–

PMNs polymorphonuclear leukocytes, *FCER1A* α subunit of the high-affinity IgE receptor, *CPA3* carboxypeptidase A3, *HDC* histidine decarboxylase, CCR2 C-C motif chemokine receptor 2, MFI mean fluorescence intensity

P values were Bonferroni-corrected, and *P*-value < 0.0026 was accepted as significant. Statistically significant P values are presented in boldface

^a^In 30 patients, we measured serum chemokines and tryptase, and in 26 patients, we measured blood absolute cell counts and gene expression (of those 41 patients, 15 were in both subgroups); serum samples from 8 patients were used for in vitro migration experiments

^b^In 20 healthy subjects, we measured serum chemokines and tryptase; basophils from 8 healthy subjects were used for in vitro migration experiments

#### Eotaxin chemokines CCL11/eotaxin, CCL24/eotaxin-2 and CCL26/eotaxin-3

There were no differences in CCL11 (median 200, 211 and 160 pg/ml), CCL24 (1124, 946 and 1159 pg/ml) and CCL26 (1903, 1898 and 1877 pg/ml) between the acute and convalescent time points and the healthy subjects (Fig. 1 and Table 2).

#### Chemokines CCL5/Rantes, CCL17/TARC, CCL21/SLC and CCL22/MDC

There were no differences in CCL5 (median 40.9, 41.8 and 47.0 ng/ml), CCL17 (342, 303 and 344 pg/ml), CCL21 (1179, 1099 and 938 pg/ml) and CCL22 (666, 717 and 766 pg/ml) between the acute and convalescent time points and the healthy subjects (Fig. 1 and Table 2).

### Increase in serum CCL2 levels highly correlates with an increase in serum tryptase

The median concentration of serum tryptase was significantly higher during the anaphylactic episode (12.9 µg/l) than later or in healthy subjects (5.4 and 3.3 µg/l, respectively; *P* ≤ 0.0005; Fig. 1 and Table 2). The median rate of increase of serum tryptase was 133.8% (range: − 6.6% to 893.4%; increase in 28/30 patients), which was comparable to the increase in serum CCL2 levels (Fig. 2). There was a significant positive correlation between the rates of increase of serum CCL2 and tryptase (r = 0.68, *P* < 0.0001) and between the acute concentration of serum CCL2 and the acute concentration of serum tryptase (r = 0.77, *P* < 0.0001; Fig. 2).

### Anaphylactic episodes involve a substantial reduction in circulating basophils

#### Basophils, monocytes, lymphocytes, and PMNs

**Fig. 3:**
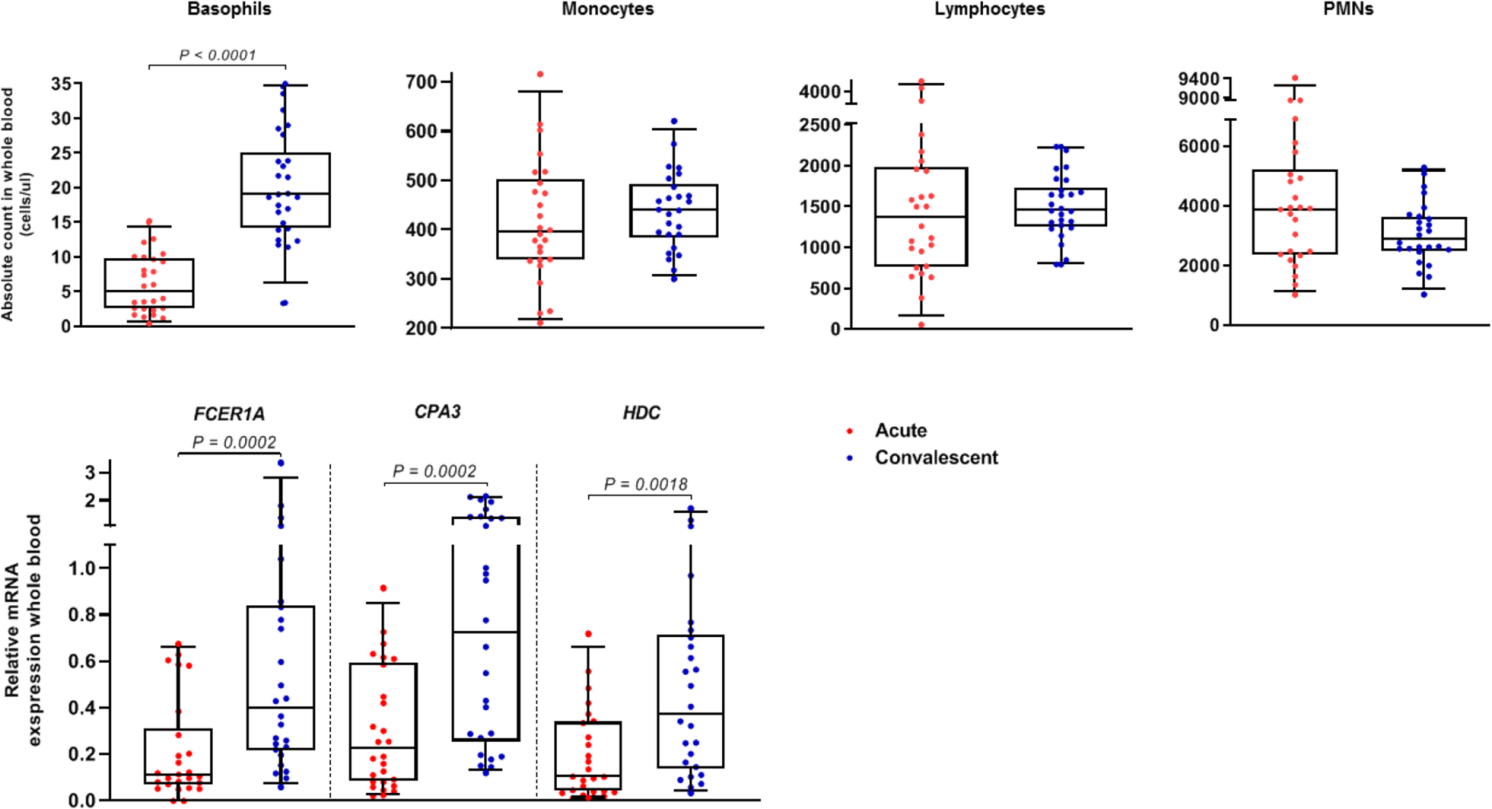
Absolute basophil, monocyte, lymphocyte and PMN counts and whole blood *FCER1A*, *CPA3*, and *HDC* gene expression in 26 patients during the acute anaphylactic episode and in convalescent samples collected later. PMNs polymorphonuclear leukocytes, *FCER1A* α subunit of the high-affinity IgE receptor, *CPA3* carboxypeptidase A3, *HDC* histidine decarboxylase

The absolute number of circulating basophils was significantly lower during the anaphylactic episode (median 5.0 cells/µl) than in convalescent blood samples collected later (19.1 cells/µl, *P* < 0.0001; Fig. 3 and Table 2). This decrease (median 65.6%, range 30.0–98.6%) was evident in all patients. There were no significant differences in the monocyte (median 396 and 441 cells/µl), lymphocyte (1378 and 1461 cells/µl), and PMN (3881 and 2875 cells/µl) absolute cell counts during the acute episode compared to convalescent blood samples collected later (Fig. 3).

#### Gene expression of basophil markers

We observed significantly lower whole blood gene expression of *FCER1A*, *CPA3*, and *HDC* during the anaphylactic episode than in convalescent samples (respectively; *P* ≤ 0.0018; Fig. 3 and Table 2). Acute whole blood gene expression of *FCER1A*, *CPA3*, and *HDC* significantly correlated with the acute absolute number of circulating basophils (r = 0.53, 0.79 and 0.81, respectively; *P* ≤ 0.005; Additional file 1: Fig. S1).

### Anaphylactic serum enhances the in vitro migration of basophils via CCL2-dependent chemotactic activity

A basophil migration assay was performed using MACS-separated human basophils and a modified Boyden chamber, with quantification by absolute flow cytometry basophil counts. Basophils from all healthy donors showed high surface expression of CCR2 (Table 2). All experiments were performed independently in triplicate (detailed information is provided in Additional file 1).

#### Basophil migration induced by HBSS and rCCL2

**Fig. 4:**
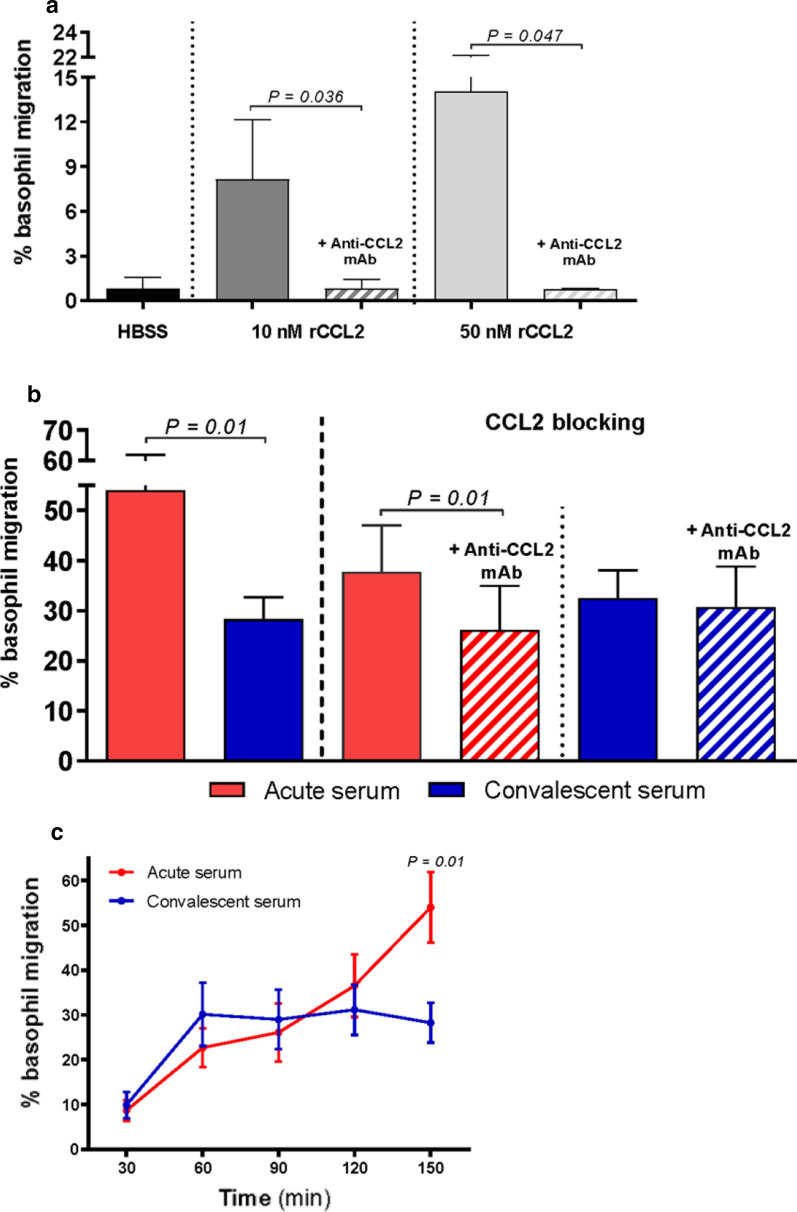
**a**–**c** Effects of CCL2 and acute and convalescent serum from patients with an anaphylactic episode on basophil in vitro migration. Basophil migration after 150 min of incubation: **a** in the presence of rCCL2 in the lower wells and after CCL2 blocking and **b** in the presence of acute or convalescent serum in the lower wells and after CCL2 blocking. **c** Time course of basophil migration (after 30, 60, 90, 120, and 150 min of incubation) in the presence of acute and convalescent serum in the lower wells. All experiments were independently performed in triplicate, with **a** basophils from 5 healthy donors; **b** basophils from the same 5 healthy donors and serum samples from 5 patients and **c** basophils from 3 healthy donors and serum samples from 3 patients (details in Additional file 1: Table S1). HBSS Hank’s balanced salt solution

As shown in Fig. 4a, during a 150-min incubation time with Hank’s balanced salt solution (HBSS), only a small number of basophils (mean, SD), 0.8 ± 0.7%) transmigrated across the polycarbonate membrane. Basophil migration was significantly enhanced by the presence of 10 nM rCCL2 and 50 nM rCCL2 (8.1 ± 4.0% and 14.0 ± 8.1%; 150 min of incubation); however, after blocking with anti-CCL2 mAbs, rCCL2-induced migration was almost completely inhibited (0.8 ± 0.6%, *P* = 0.036 and 0.8 ± 0.1%, *P* = 0.047, blocking at 10 nM and 50 nM of rCCL2, respectively).

#### Basophil migration induced by anaphylactic and convalescent serum

We compared the effects of acute serum collected during the anaphylactic episode and convalescent serum collected later (Table 2) on basophil migration. After 150 min of incubation, the number of transmigrated basophils was significantly higher (twofold) in the presence of acute serum in the lower wells than in the presence of convalescent serum in the lower wells (mean, SD: 54.0 ± 19.3% vs. 28.3 ± 11.7%, respectively; *P* = 0.01) (Fig. 4b). The number of transmigrated cells increased linearly until 150 min of incubation and then plateaued (Fig. 4c). A neutralizing antibody against CCL2 significantly blocked basophil transmigration induced by anaphylactic serum (37.7 ± 20.8% vs. 26.1 ± 19.7%; *P* = 0.001). However, a neutralizing antibody against CCL2 did not show any blocking effect on the chemotactic response induced by convalescent serum (32.5 ± 12.5% vs. 30.7 ± 18.3%) (Fig. 4b).

## Discussion

Our study demonstrated that there is a selective and significant increase in serum CCL2 chemokine levels during anaphylactic reactions. No significant changes were observed for other chemokines, which were previously implicated in different allergic diseases or during allergic inflammation [15–25]. Thus, the chemokine patterns of CCL5, CCL7, CCL8, CCL11, CCL13, CCL17, CCL21, CCL22, CCL24, and CCL26 might be of limited importance for anaphylaxis. While activation of the pathways associated with multiple inflammatory cytokines, including IL-2, IL-6, IL-10, and TNFRI, were observed during anaphylaxis [6, 9], the selective CCL2 chemokine cascade might be important for chemotactic activity during anaphylactic reactions. It was recently reported that the CCL2-FcεRI-histone deacetylase 3 signalling axis mediates passive anaphylaxis by increasing the expression of CCL2 in mast cells [28, 29]. After allergen stimulation, histone deacetylase 3 binds to FcεRI, and histone deacetylase 3 increases the expression of SP1 and C-Jun, which bind to the promoter sequences of CCL2 to increase the expression of CCL2 [28, 29]. Conversely, in the absence of allergen stimulation, histone deacetylase 3 binds to the promoter sequences of CCL2 to suppress the expression of CCL2 [28].

Our study design and the median time between symptom onset and sample collection were highly comparable with those of previous studies of anaphylaxis [3, 6, 7, 9, 30]. This suggests that this is an unbiased comparison, including the confirmation that the chemokines CCL5 and CCL11, which may affect other important effector cells of allergic inflammation such as eosinophils, are not induced during anaphylaxis [6, 7]. The nature of the management of anaphylaxis (including administration of high-dose corticosteroids) makes it difficult to exclude the potential confounding effects of treatment. However, it was previously demonstrated that serum CCL2, CCL5, and CCL11 levels are not affected by corticosteroid treatment [7]. Nevertheless, corticosteroid treatment or the time between symptom onset and sample collection may have affected the levels of the other measured chemokines.

In the present study, we could not determine the cellular sources of CCL2 during anaphylaxis. However, we showed a highly significant positive correlation between elevated blood levels of acute CCL2 and tryptase and strong similarities between the percentage increases of CCL2 and tryptase from convalescent serum samples. Tryptase is largely mast cell-derived, and mature β-tryptase is stored in mast cell granules and released upon mast cell activation during acute anaphylaxis [2, 5–7]. Therefore, it is tempting to speculate that the blood CCL2 increase that occurs during anaphylaxis is IgE related and mainly mast cell derived. In vitro activation of human mast cells by anti-IgE induces secretion of CCL2 after 30 min of stimulation, and this increase is observed earlier than TNF-α, IL-13, or GM-CSF mast cell secretion, peaking 90 min after anti-IgE stimulation [31]. Furthermore, mast cell CCL2 secretion is induced at low allergen concentrations or in the context of low receptor occupancy with IgE [32], and due to the short time frame within which the increase in serum CCL2 was observed in the current and previous studies [7], rapid induction of CCL2 production by the allergen-IgE axis might be feasible. Nevertheless, CCL2 is produced by a variety of cell types, including endothelial cells and smooth muscle cells [33], astrocytes as a major source of CCL2 during central nervous system inflammation [34] and cancer and stromal cells as a major source of CCL2 in tumour microenvironments [35]; thus, the cellular sources of CCL2 during anaphylaxis are currently unknown. Additional and substantially studies of mast cell CCL2 expression and secretion are required to confirm this speculation.

The results of experimental allergen challenge in the nose, airways, and skin have demonstrated that there is an influx of basophils to inflammatory sites several hours after allergen exposure [36–39]. Furthermore, it was recently demonstrated that airway allergen challenge induces TLR-dependent CCL2 production in the lungs and that circulating basophils are recruited to the lungs by CCL2-mediated transendothelial migration and a chemotactic gradient [40]. Our study demonstrated significantly enhanced CCL2-mediated chemotactic activity of anaphylactic serum for the migration of isolated basophils. Conversely, no CCL2-mediated basophil chemotactic activity was observed for the convalescent serum collected later after the reaction. The CCL2-mediated basophil chemotactic activity of anaphylactic serum and the reduction in circulating basophils during anaphylaxis, coupled with the previous finding that basophils are the granulocytes most resistant to apoptosis [41], suggest that anaphylaxis induces rapid CCL2-mediated basophil migration rather than elimination by apoptosis. Moreover, anaphylaxis-related basophil migration appears to be selective because no significant changes were observed for monocytes, lymphocytes, PMNs, or chemokines that may affect other effector cells, and the decreased gene expression of basophil markers *FCER1A*, *CPA3* and *HDC* corroborates the flow cytometry data. Therefore, it is tempting to speculate that CCL2 might be mast cell derived and that circulating basophils influx to the sites where the activation and degranulation of mast cells occur, thereby contributing to the clinical presentation of the sites of allergic reaction. This is consistent with clinical observations of different severities and end-organ patterns of anaphylaxis, which suggest that local rather than generalized mast cell and/or basophil degranulation may predominate in some individuals [41]. However, a substantially broader assessment is needed to explore and confirm CCL2-mediated cellular crosstalk during acute allergic reactions.

Although CCL2 displays major chemotactic activity for monocytes and interferes with the egress of monocytes from the bone marrow to the circulation during homeostasis and inflammation [42], no changes in circulating monocytes were observed during anaphylaxis. However, studies in mice have shown that depletion of monocytes/macrophages can reduce anaphylaxis in both IgG-mediated passive models and active models [2, 11].

## Conclusion

In conclusion, our data suggest that during anaphylaxis, an increase in the chemokine CCL2 occurs, which correlates with CCL2-mediated chemotactic activity in basophils and substantial migration of circulating basophils. Our findings imply an important and specific role for CCL2 in the pathophysiology of human anaphylaxis.

## Supplementary information

**Additional file 1.** Additional methods and results. **Table S1.** Detailed information on the number of participants for whom we assessed different laboratory parameters. Additional results. **Figure S1.** Correlation between absolute basophil counts and whole-blood *FCER1A*, *CPA3*, and *HDC* gene expression in 26 anaphylactic patients

## Data Availability

Raw data were generated at the University Clinic of Respiratory and Allergic Diseases, Golnik, Slovenia. Derived data supporting the findings of this study are available from the corresponding author upon reasonable request.

## Abbreviations

CCL: C-C Motif Chemokine Ligand
CCR: C-C motif chemokine receptor
CPA: Carboxypeptidase A3
FCER1A: α subunit of the high-affinity IgE receptor
HBSS: Hank’s balanced salt solution
HDC: l-Histidine decarboxylase
MCP: Monocyte chemoattractant protein
MDC: Macrophage-Derived Chemokine
PMNs: Polymorphonuclear leukocytes
SLC: Secondary Lymphoid Tissue Chemokine
TARC: Thymus and Activation Regulated Chemokine
