# Supplementary material for: Chemokines during anaphylaxis: the importance of CCL2 and CCL2-dependent chemotactic activity for basophils
Source: Clin Transl Allergy. 2020 Dec 15;10:63. doi: 10.1186/s13601-020-00367-2 (PMC7737350; doi:10.1186/s13601-020-00367-2)
Supplement: Supplementary file 1 — Additional file 1. Additional methods and results. Table S1. Detailed information on the number of participants for whom we assessed different laboratory parameters. Additional results. Figure S1. Correlation between absolute basophil counts and whole-blood FCER1A, CPA3, and HDC gene expression in 26 anaphylactic patients [file 13601_2020_367_MOESM1_ESM.docx]

**Additional file 1**

CHEMOKINES DURING ANAPHYLAXIS: THE IMPORTANCE OF CCL2 AND CCL2-DEPENDENT CHEMOTACTIC ACTIVITY FOR BASOPHILS

Romana Vantur^1^, Marusa Rihar^1^, Ana Koren^1^, Matija Rijavec^1,2^, Peter Kopac^1,3^, Urska Bidovec-Stojkovic^1^, Renato Erzen^1,3^, Peter Korosec^1^

^1^University Clinic of Respiratory and Allergic Diseases, Golnik, Slovenia.

^2^Biotechnical Faculty, University of Ljubljana, Ljubljana, Slovenia.

^3^Medical Faculty, University of Ljubljana, Ljubljana, Slovenia.

Correspondence: Peter Korosec, University Clinic of Respiratory and Allergic Diseases Golnik, Golnik 36, 4204 Golnik, Slovenia, E-mail: peter.korosec@klinika-golnik.si

Running title: Chemokines during anaphylaxis

**Additional methods and results**

**Serum chemokine levels**

We measured serum concentrations of chemokines CCL2, CCL7, CCL5, CCL11, CCL17, CCL22, CCL24 (Quantikine Human Immunoassay; R&D Systems, Minneapolis, MN, USA), CCL8, CCL13 (Biolegend, San Diego, California, CA, USA), CCL21 and CCL26 (Abnova, Taipei, Taiwan), by using different ELISA tests according to manufacturer’s instructions.

**Absolute cell count**

Absolute cell counts were performed on the heparinized whole blood as previously described [1,2]. A precise volume of whole heparinized blood (50 µl) was incubated for 20 min with PE-conjugated anti-CD123 and PerCP-conjugated anti–HLA-DR mAbs (both BD Biosciences, San Jose, CA, USA). Whole blood samples were then lysed, washed, and fixed. For the absolute basophil count (CD123⁺, and HLA-DR⁻ cells), 50 µl of AccuCount Fluorescent microbeads (7.7 mm, 51,011 particles per 50 mL; Spherotec Inc., Libertyville, IL, USA) was added to the fixed samples before flow cytometric analysis. All samples were analyzed within 2 hours on a FACS Canto 2 flow cytometer (BD Biosciences). Leukocytes were gated according to lysed whole-blood forward-scatter/side-scatter characteristics. Absolute numbers of basophils, monocytes, lymphocytes, and PMNs per microliter of whole blood were calculated by using the following equation:

*Absolute cell number = (Number of cells / Number of events per microbead region) x (Number of microbeads used in test / Volume of the whole blood sample).*

**CCR2 surface expression on basophils**

50 µl of heparinized whole blood was incubated for 20 min with PE-conjugated anti-CD123, PerCP-conjugated anti–HLA-DR (both BD Biosciences), and APC-conjugated anti-CCR2 antibody mAb (CD192; Miltenyi Biotec, Bergisch Gladbach, Germany) and then lysed, washed, and fixed. All samples were analyzed within 2 hours on a FACS Canto 2 flow cytometer (BD Biosciences). Basophils were identified as low side-scatter, CD123⁺, and HLA-DR2⁻ cells[1,2], and CCR2 surface expression on basophils was quantified as mean fluorescence or % of positive cells.

**Gene expression of blood basophil markers**

We analyzed gene expression of the α-subunit of the high-affinity IgE receptor (*FCER1A*, Hs00175232_m1), carboxypeptidase A3 (*CPA3*, Hs00157019_m1), and L-histidine decarboxylase (*HDC*, Hs00157914_m1) as previously described.[1,2] Total RNA was isolated by using the PAXgene Blood miRNA Kit (PreAnalytiX) and quantified using NanoDrop 2000 (Thermo Fisher). Reverse transcription reactions were performed using the High Capacity cDNA Reverse Transcription Kit (Applied Biosystems), followed by quantification of cDNA by using real-time PCR (ABI PRISM 7500 Real-Time PCR System; Applied Biosystems, Foster City, Calif) at standard conditions with TaqMan Universal PCR Master Mix (Thermo Fisher). Expression levels were normalized against ribosomal 18s RNA Endogenous Control (Thermo Fisher). All measurements were performed in triplicates for each sample and time point, and relative expressions were analyzed using the ∆∆ cycle threshold method [3].

***In vitro* migration assays**

**Basophil isolation**

Basophils were isolated from peripheral blood by negative immunomagnetic selection. First, we isolated peripheral blood mononuclear cells (PBMNC) from heparinized whole blood by performing gradient separation with Lympholyte-H (Cedarlane, Canada). Briefly, 40 ml of fresh peripheral whole blood per healthy donor was diluted with 3.5 times of PBS volume (Immuno Concepts Inc., California, USA). 15 ml of Lympholyte-H was placed in a 50 ml centrifuge tube and 35 ml of diluted blood was carefully layered on it. We centrifuged 30 min 1400 rpm/min at room temperature and without a break, carefully transferred the middle layer of PBMNCs in a new 50 ml centrifuge tube, and added PBS to a final volume of 50 ml. We centrifuged 7 min 1200 rpm/min, discarded the supernatant, resuspended in 35 ml of PBS, and centrifuged 7 min 1000 rpm/min. Again, the supernatant was discarded and the remaining pellet was resuspended in 10 ml of PBS. 50 µL of the resuspended pellet was pipetted away for the cell count, whereas the remaining volume of the pellet was centrifuged 7 min 1200 rpm/min and supernatant discarded.

The next step was basophil purification using negative selection with immunomagnetic beads. PBMNCs (10^7^ cells) were resuspended in 2 ml of MACS buffer (Miltenyi Biotec, Bergisch Gladbach, Germany) and centrifuged 10 min 1200 rpm/min. Cells were resuspended in 30 µl of MACS buffer. 10 µl of FcR blocking reagent (Miltenyi Biotec) and 10 µl of the cocktail of biotin-labeled antibodies (Miltenyi Biotec) were added. After incubation, 10 min at 8 °C, 30 µL of MACS buffer, and 20 µl of anti-biotin microbeads (Miltenyi Biotec) were added. We incubated for another 15 min at 8 °C, washed cells with 2 ml of MACS buffer, and centrifuged 7 min 1200 rpm/min. The supernatant was discarded and cells were resuspended in 500 µl of MACS buffer. Magnetic separation was finally performed, using MidiMACS™ separator. Separation column size LS (Miltenyi Biotec), was first washed with MACS buffer and 500 µl of PBMNC cell suspension was applied. Washing with MACS buffer was repeated 3 times and after separation, basophils represented an eluted cell fraction of unlabelled collected cells. We centrifuged cell fraction 7 min 1200 rpm/min and discarded supernatant. Cells were resuspended in 1 ml of MACS buffer. 50 µL of resuspended cells was pipetted away for the cell count. The remaining cell volume was centrifuged 7 min 1200 rpm/min, the supernatant discarded, and cells resuspended in an appropriate volume of Hank’s balanced salt solution (HBSS, Gibco, California, USA).

**Basophil migration experiments**

Isolated cells were first counted in a counting chamber (hemocytometer). According to the cell count, we resuspended cells in HBSS (Gibco, California, USA) to obtain roughly 2x10^5^ cells per ml. The absolute basophils count was performed by flow cytometry for every isolation (as described previously[1,2] and above) to calculate and adjust the absolute number of seeded basophils. For every experiment, we sedded 2x10^4^ basophils. All experiments were performed in triplicates.

Experiments with recombinant CCL2

For initial basophil migration experiments with recombinant CCL2 (rCCL2) and neutralizing anti-CCL2 antibody, 5 out of 8 healthy donors (3 males, 2 females; average age, 28 years; range, 25-39) were recruited. We isolated basophils from the peripheral blood of healthy volunteers as described above. Modified Boyden chambers (polycarbonate membrane cell culture inserts of pores size 5.0 µm) were used for basophil chemotaxis assay. We placed in the upper part of the chamber (insert) 100 µl of basophil cell suspension with 2x10^4^ cells and 10 µl of autologous serum of healthy donors. In the lower part of the chamber (well of 24-well cell culture plate) 550 µl of 50 nM or 10 nM rCCL2 in HBSS solution or 550 µl of HBSS buffer was added. Anti-CCL2 neutralizing antibody (in ratio 1:10) was used to block rCCL2 in the HBSS solution (blocking time: 30 minutes) and the same volumes and concentrations of rCCL2 solution were used. Plates were incubated at 37 °C and 5 % CO_2_ for 150 min. Median numbers (ranges) of migrated and collected basophils were as follows: 144 (38-322) basophils in HBSS solution, 1948 (1798-4670) basophils in 50 nM rCCL2, 154 (146-166) basophils in 50 nM rCCL2 + anti-CCL2, 1236 (1092-2548) basophils in 10 nM rCCL2 and 134 (70-292) basophils in 10 nM rCCL2 + anti-CCL2.

Basophil migration against serum and anti-CCL2 blockade

In the second part, we tested basophil migration against acute and convalescent serum and the effect of neutralizing antibodies against CCL2. Isolated basophils from 3 out of 8 healthy donors (2 females and 1 male; average age, 33 years; range, 27-44), and serum samples (acute (median CCL2 (IQR) 1726 (1381) pg/ml) and convalescent samples (230 (94) pg/ml)) from 3 patients (2 males and 1 female; average age, 64 years; range, 53-73) with severe, grade IV reactions, were recruited. Again, modified Boyden chambers were used and in the upper part of the chamber, 100 µl of basophil cell suspension with 2x10^4^ cells and 10 µl of convalescent serums were placed. In the lower part, 500 µl of HBSS buffer and 50 µl of acute or convalescent serum was added. We incubated at 37 °C and 5 % CO_2_ for 30, 60, 90, 120, and 150 min. Median numbers (ranges) of migrated and collected basophils against acute samples were as follows: 1422 (118-3788) after 30 min, 3660 (1536-7948) after 60 min, 3418 (1414-13140) after 90 min, 8306 (1802-13242) after 120 min, and 12579 (5562-13980) basophils after 150 min. Median numbers (ranges) of migrated and collected basophils against convalescent samples were as follows: 1360 (592-5774) after 30 min, 4016 (2206-12306) after 60 min, 5476 (846-12848) after 90 min, 5278 (2606-13046) after 120 min, and 5840 (3176-9560) basophils after 150 min.

For an investigation of the effect of neutralizing antibody against CCL2 on acute and convalescent serum, basophils were also isolated, counted, and seeded as described above. The same five healthy donors as in the initial part of basophil migration experiments were recruited. Serum samples (acute (median CCL2 (IQR) 2201 (2158) pg/ml) and convalescent samples ( (265 (320) pg/ml)) from 5 patients (4 males and 1 female; average age, 55 years; range, 37-76) with grade IV (2 patients), grade III (2 patients) and grade I reaction (1 patient), were recruited. Modified Boyden chambers were used and the experiment was conducted as described before. The only difference was the addition of neutralizing anti-CCL2 antibody (ratio 1:10) to acute and convalescent serums, to determine the impact of the blockade. Incubations were done at 37 °C and 5 % CO_2_ for 150 min. Median numbers (ranges) of migrated and collected basophils were as follows: 8980 (2800-12160) basophils against acute serum and 5060 (1200-9260) basophils after CCL2 blockade in acute serum, 7360 (3580-9600) basophils against convalescent serum, and 6740 (2040-10540) basophils after CCL2 blockade in convalescent serum.

**Basophil collection and calculation of migration**

After incubation, inserts (upperparts with previously added 2x10^4^ basophils) were removed from the 24-well cell culture plates and contents of lower parts were collected and transferred to the flow cytometer tubes for staining, quantifying, and analyzing with flow cytometry. Each well was additionally washed with PBS buffer and centrifuged, 5 min 1600 rpm/min. The absolute basophils count was performed by flow cytometry as described above (to calculate the absolute number of migrated basophils).

Basophil migration was calculated by using the following equation:

*Basophil migration (%) = (absolute number of migrated basophils / absolute number of seeded basophils) x 100*

*The absolute number of migrated basophils* represents the number of basophils that were collected from the lower part of the Boyden chamber. *The absolute number of seeded basophils* represents the number of basophils that were seeded in the upper part of the chamber (2x10^4^ cells).

**References**

1. Čelesnik N, Vesel T, Rijavec M, Šilar M, Eržen R, Košnik M, et al. Short-term venom immunotherapy induces desensitization of FcεRI-mediated basophil response. Allergy Eur J Allergy Clin Immunol. 2012.;67:1594–600. doi:10.1111/all.12044

2. Korosec P, Turner PJ, Silar M, Kopac P, Kosnik M, Gibbs BF, et al. Basophils, high-affinity IgE receptors, and CCL2 in human anaphylaxis. J Allergy Clin Immunol. 2017.;140. doi:10.1016/j.jaci.2016.12.989

3. Livak KJ, Schmittgen TD. Analysis of Relative Gene Expression Data Using RealTime Quantitative PCR and the 2 ∆∆ CT Method. 2001;408:402–408. doi:10.1006/meth.2001.1262


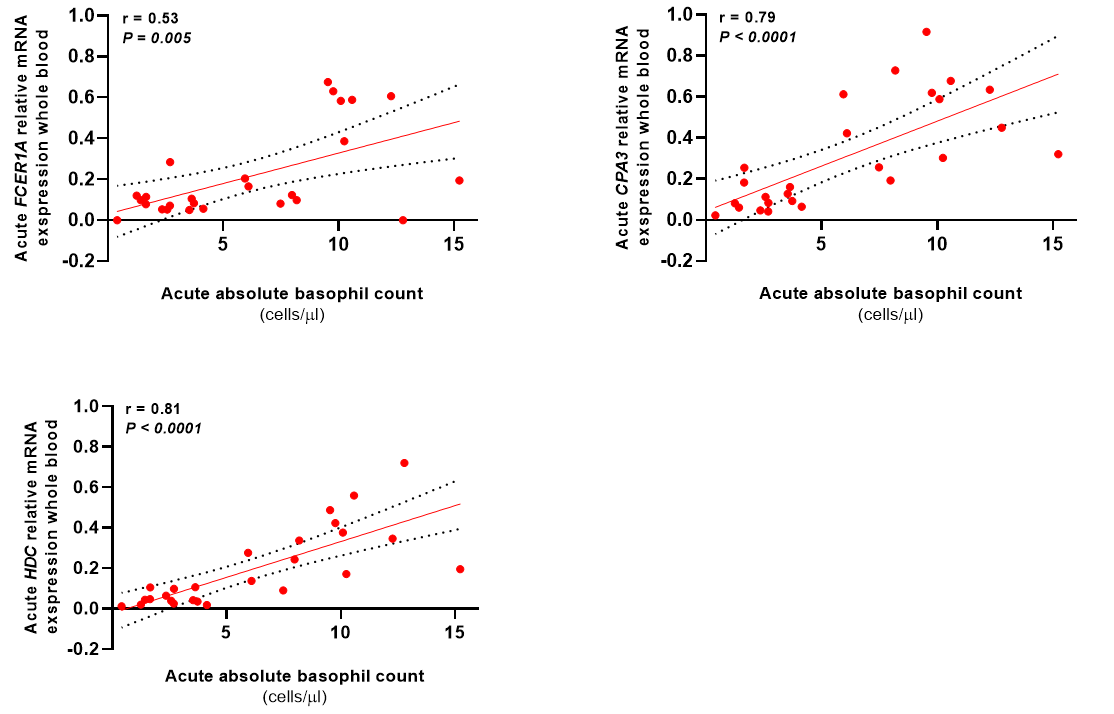


**Fig S1**. Correlation between absolute basophil counts, and whole-blood *FCER1A*, *CPA3*, and *HDC* gene expression in 26 patients with acute anaphylactic episodes.

**Table S1.** Detailed information on the number of participants for whom we assessed different laboratory parameters.

|  | **Serum chemokines** | **Serum CCL2 only** | **Serum tryptase** | **Absolute cell counts (basophils, monocytes, lymphocytes, and PMNs)** | **Gene expression in whole blood (*FCER1A, CPA3, and HDC*)** | **Absolute basophil count and CCR2 expression** | ***In vitro* migration assays** | | |
| --- | --- | --- | --- | --- | --- | --- | --- | --- | --- |
|  |  |  |  |  |  |  | **rCCL2**  **and**  **CCL2 blocking** | **Anaphylactic serum and**  **CCL2 blocking** | **Time course of basophil migration** |
| **Patients with an acute episode of anaphylaxis (n=49)** | | | | | | | | | |
| Serum chemokines and blood cells (n=41) | 30 | / | 30 | 26 | 26 | / | / | / | / |
| Seruma of patients used for *in vitro* migration assays (n=8) | / | 8 | 8 | / | / | / | / | 5 | 3 |
| **Healthy subjects (n=28)** | | | | | | | | | |
| Serum chemokines (n=20) | 20 | / | 20 | / | / | / | / | / | / |
| Basophils of healthy donors used for the *in vitro* migration assays (n=8) | / | / | / | / | / | 8 | 5 | 5 | 3 |
